# Development of ^99m^Tc-cefadroxil loaded chitosan nanoparticles as a novel radiotracer for detection of septic and aseptic foci

**DOI:** 10.1186/s11671-026-04930-x

**Published:** 2026-10-13

**Authors:** Marwa S. El Refaye, Safaa B. Challan, H. E. Ramadan, S. I. Khater

**Affiliations:** 1 Radioactive Isotopes and Generators Department, Hot Laboratories and Waste Management Center, Egyptian Atomic Energy Authority P.O.Box, 13759 Cairo, Egypt; 2 Cyclotron Facility, Nuclear Research Center, Egyptian Atomic Energy Authority P.O.Box, 13759 Cairo, Egypt; 3 Labeled Compounds Department, Hot Laboratories and Waste Management Center, Egyptian Atomic Energy Authority, P.O.Box, 13759 Cairo, Egypt

**Keywords:** Cefadroxil, Chitosan nanoparticles, Technetium-99m, Septic foci, Aseptic foci, XRD, TEM, FTIR, Zeta potential

## Abstract

Early detection of inflammation is critical for preventing disease progression, and nuclear medicine plays a pivotal role in this context. Imaging modalities such as single-photon emission computed tomography (SPECT) enable the early identification of functional abnormalities through the use of targeted radiotracers that specifically localize inflammatory processes. In this regard, antibiotic-loaded nanoparticles have emerged as a promising platform for inflammation imaging, integrating diagnostic capabilities with potential therapeutic functionality. Among these systems, chitosan-based nanocarriers (CS-NPs), a nano-polymeric pharmaceutical excipient, were employed for the encapsulation of the antibiotic cefadroxil (CDX). The formulation was prepared via a simple ionic gelation (cross-linking) technique. The physicochemical properties of the resulting nanoparticles were characterized using transmission electron microscopy (TEM), Fourier-transform infrared spectroscopy (FT-IR), zeta potential analysis, and X-ray diffraction (XRD). Drug encapsulation efficiency and loading capacity were subsequently quantified by UV–visible spectrophotometry. For imaging applications, CDX-loaded CS-NPs (400 µg) were radiolabeled with technetium-99m in the presence of stannous chloride (50 µg) as a reducing agent, yielding a complex with a radiochemical efficiency of 94 ± 1.3%. The resulting 99mTc–CDX–CS-NPs demonstrated excellent in vitro stability in fresh serum for up to 6 hours, which is suitable for diagnostic imaging applications. In vivo biodistribution studies in murine models of both septic and aseptic inflammation revealed significant tracer accumulation at infected and inflamed sites, with high target-to-non-target (T/NT) ratios of 8.55 and 6.02 after 2 hours post-injection, respectively. These findings suggest the developed radiotracer's ability to effectually localize septic and aseptic foci, irrespective of their underlying etiology.

## Highlights

Chitosan nanoparticles were produced for cefadroxil encapsulation via ionic cross-linking.

Characterization confirmed successful nanoparticle formation.

CDX-CS-NPs were efficiently radiolabeled with 99mTc offering a radiochemical yield: 94 ± 1.3%.

The radiolabeled nanoparticles demonstrated good serum stability for up to 6 h.

Significant accumulation in septic and aseptic inflammation models was observed in murine models.

High T/NT ratios (6.02 and 8.55 at 2 h) indicate strong potential for septic and aseptic foci imaging.

## Introduction

Inflammation is a complex biological response of the body’s tissues to exogenous harmful stimuli, including pathogens, damaged cells, and irritants [1]. An insufficient inflammatory response may permit the progression of tissue damage caused by such stimuli, particularly microbial infections. Conversely, excessive or chronic inflammation is implicated in the pathogenesis of numerous diseases, including allergic conditions, cancer, atherosclerosis, and osteoarthritis. Emerging evidence suggests that cancer-related inflammation (CRI) may play a pivotal role in promoting genomic instability and the accumulation of random genetic alterations in melanoma cells [2]. Accordingly, early and accurate detection of inflammatory foci is critical, as it may prevent progression to chronic inflammation and enhance clinical outcomes. This is especially relevant in conditions such as implant-associated infections, where timely intervention can reduce healing time and minimize severe complications, including tissue necrosis [3,4].

*Staphylococcus aureus (S. aureus)* is a genus of Gram-positive bacteria characterized by spherical (cocci) cells that commonly colonize the skin and mucosal surfaces of humans and other warm-blooded animals as part of the normal microbiota. Although many staphylococcal species exist as harmless commensals, certain species—most notably *Staphylococcus aureus*—are clinically significant opportunistic pathogens. *S. aureus* is capable of causing a broad spectrum of infections, ranging from superficial skin and soft tissue infections to severe, life-threatening conditions such as pneumonia, bacteremia, and toxic shock syndrome. Furthermore, the emergence of antibiotic-resistant strains, particularly methicillin-resistant *S. aureus* (MRSA), represents a major public health concern due to limited therapeutic options and increased morbidity and mortality [5].

Cefadroxil (CDX) is a first-generation cephalosporin antibiotic with potent activity against Gram-positive bacteria, including *Staphylococcus aureus* [6,7]. In parallel, chitosan—a biocompatible and biodegradable polysaccharide derived from chitin—is widely employed in nanoparticle formulation due to its favorable properties, including muco-adhesiveness, anti-inflammatory activity, and low immunogenicity [8,9]. Owing to these characteristics, chitosan is recognized as an effective biopolymer for diverse biomedical applications, including green chemistry approaches, anticancer strategies, and wound-healing systems [10].

Encapsulation of CDX within chitosan nanoparticles (CDX-CSNPs) has been shown to enhance drug entrapment efficiency and improve targeted delivery to inflamed tissues. Accordingly, recent studies have focused on developing in situ gel systems incorporating CDX-loaded chitosan nanoparticles, which demonstrate promising wound-healing potential by effectively suppressing associated bacterial infections [11].

Nanoparticles have recently garnered considerable attention as theragnostic platforms in the biomedical field, providing an effective means for simultaneous diagnosis and treatment of human diseases. Their unique physicochemical properties enable enhanced disease characterization, targeted drug delivery, and improved therapeutic outcomes. In particular, chitosan-based nanomaterials have demonstrated significant contributions across biomedicine and pharmaceutical sciences, owing to their biocompatibility, biodegradability, and versatile functionalization potential, thereby supporting a wide range of applications [12].

The emergence of antimicrobial resistance represents a global challenge that necessitates the development of more effective, safer, and synergistic therapeutic alternatives. These concerns have driven growing interest in applying nanotechnology to antibacterial research, offering a promising strategy to combat resistant bacterial strains. The integration of nanomaterials in this field is attributed to their unique physicochemical properties, including tunable size and morphology, and their ability to co-deliver multiple active agents, thereby enabling targeted and enhanced antimicrobial activity. Consequently, nanomaterials have emerged as a promising alternative to conventional antibiotics in a wide range of medical applications [13].

Previous studies have investigated the application of antibiotic-loaded nanoparticles for inflammation imaging and the detection of infection sites. These approaches include localized antimicrobial delivery via nanocarriers, which address limitations such as antimicrobial resistance and systemic toxicity while enhancing targeted drug release at sites of infection [14]. A comprehensive review has also highlighted the use of polymeric, lipid-based, mesoporous silica, and inorganic nanocarriers for levofloxacin encapsulation, demonstrating improved controlled-release profiles and enhanced antibacterial efficacy [15]. Furthermore, a systematic review examined the incorporation of gentamicin and vancomycin into various nanoscale drug delivery systems, discussing the challenges posed by conventional therapies and the potential of nanoencapsulation to improve pharmacokinetic and pharmacodynamic properties [16]. Additional studies have reported enhanced efficacy of ciprofloxacin when conjugated with silver nanoparticles, attributed to improved cellular penetration, disruption of bacterial biofilms, and sustained drug release [17].

This study aims to investigate the potential enhancement of cefadroxil’s antimicrobial efficacy by formulating it into chitosan-based nanoparticles. Cefadroxil-loaded chitosan nanoparticles (CDX-CSNPs) were synthesized, followed by radiolabeling with technetium-99m, with systematic evaluation of the parameters influencing radiolabeling efficiency and yield optimization. The resulting ^99m^Tc-CDX-CSNP nano formulation was subsequently assessed for its biological performance in murine models of infection and inflammation. Particular emphasis was placed on evaluating its potential biomedical applications, including its ability to target and localize inflammatory foci for early detection, given the critical importance of timely diagnosis in inflammatory pathologies.

## Materials and methods

### Materials

Cefadroxil was supplied by Pharco Pharmaceuticals (Egypt) and has a molecular formula of C₁₆H₁₇N₃O₅S with a molecular weight of 363.4 g/mol. Chitosan (CS) of medium molecular weight (200 kDa) and stannous chloride (SnCl₂) were obtained from Sigma-Aldrich (USA). Sodium tripolyphosphate (STPP) was purchased from Finar Chemicals and used without further purification. All other chemicals and reagents were of analytical grade and procured from Merck Group. Throughout all experiments, double-distilled and deoxygenated water was used. Thin-layer chromatography (TLC) was performed using aluminum sheets (20 × 25 cm) precoated with silica gel 60 F₂₅₄ (SG-60 F254) from Merck Group. A ⁹⁹Mo/⁹⁹ᵐTc generator was kindly provided by the Radioisotopes Manufacturing Facility, a division of the Atomic Energy Authority, Inshas, Egypt. Radioactivity measurements were carried out using a γ-counter supplied by Nuclear Enterprises Ltd.

### Animals

Male Swiss albino mice were used throughout the biological assessment. The mice used weighed 30 to 35 grams. The mice were obtained from the Biological Applications Department of the Nuclear Research Center of the Egyptian Atomic Energy. They were saved under fixed nutritional conditions throughout the biological experiment. They were controlled in a clean-air room at 25 ± 3°C. Water and laboratory diet were available throughout the study. This work was performed under the supervision of the animal research protocols documented by the Research Ethics Committee at the National Center for Radiation Research and Technology (REC-NCEET), affiliated with the Egyptian Atomic Energy Authority (EAEA), with Ethical Approval Number (F/33 A/25).

### Procedure for blank nanoparticles preparation

Initially, 500 mg of chitosan was dissolved in 100 mL of 2% (v/v) acetic acid to obtain a homogeneous solution. In parallel, 400 mg of sodium tripolyphosphate (STPP) was dissolved in 50 mL of deionized water. The STPP solution was then added dropwise to the chitosan solution under continuous magnetic stirring, leading to the gradual formation of a white opalescent suspension of chitosan nanoparticles via ionic gelation. The reaction mixture was subsequently stirred for an additional 2 hours to ensure complete nanoparticle formation. Thereafter, the suspension was centrifuged at 12,000 rpm for 45 minutes using an ultracentrifuge based on [18]. The supernatant was carefully discarded, and the resulting pellet was redispersed in double-distilled water. This washing step was repeated 4–5 times, with centrifugation after each cycle, to remove any unreacted STPP and residual impurities. Finally, the purified nanoparticle precipitate was collected and dried for further use [19].

### Production of Cefadroxil loaded chitosan nanoparticles

A chitosan solution was first prepared by dissolving 500 mg of chitosan in 100 mL of 2% (v/v) acetic acid under magnetic stirring at 600 rpm for 30min to obtain a homogeneous solution. Subsequently, different amount of cefadroxil (25, 50, 100) mg dissolved in 10 mL of acetone was added dropwise under continuous stirring. A sodium tripolyphosphate (STPP) solution (400 mg in 50 mL of deionized water) was then introduced gradually into the reaction mixture under constant stirring for 1h to facilitate nanoparticle formation via ionic cross-linking. The resulting suspension was centrifuged at 12,000 rpm for 45min at 25°C. The obtained pellet was redispersed in double-distilled water and washed 4–5 times to remove any unencapsulated drug, with centrifugation performed after each washing cycle. The collected supernatants were preserved for subsequent quantification of drug encapsulation efficiency using UV–visible spectrophotometry.

### Characterization of cefadroxil loaded chitosan nanoparticles

The obtained sample was characterized using X-ray diffraction (XRD) analysis performed on an X-lab Shimadzu X6000 diffractometer equipped with Cu-Kα radiation. Particle size and morphology were examined using high-resolution transmission electron microscopy (HR-TEM; Tecnai G20, FEI, Netherlands). Functional group identification was carried out by Fourier transform infrared (FTIR) spectroscopy (PerkinElmer Model 1650), within the spectral range of 4000–400 cm⁻¹ using the KBr pellet technique. In addition, the surface charge (zeta potential) of the nanoparticles was determined using a Zetasizer (Version 6.32).

### Cefadroxil loading measurements

The amount of cefadroxil (CDX) encapsulated within the nanoparticulate system was determined indirectly by quantifying the unencapsulated drug remaining in the supernatant, based on its absorbance at 240 nm [13]. A calibration curve was constructed, and sample absorbance measurements were recorded using a HITACHI U-2800 UV–visible spectrophotometer in 3 mL quartz cuvettes. Drug entrapment efficiency (EE) was defined as the percentage of drug successfully loaded into the nanoparticles. Determination of EE is essential for optimizing drug loading, minimizing drug wastage, and ensuring formulation efficiency. It is calculated according to Eq. (1) [18, 20]:

1 $${EE}=\frac{{Total}\,{CDX}\left({\_g}\right)-{Free}\,{CDX}\left({\_g}\right)}{{Total}\,{CDX}\left({\_g}\right)}\times 100$$

### Radiolabeling of CDX-CS-NPs

The radiolabeling procedure was performed under an oxygen-free atmosphere to minimize oxidation of the reducing agent and radiolabeling components. Accordingly, all reactions were conducted in evacuated 10 mL glass vials, and all solutions were purged with nitrogen gas prior to use. Since generator-eluted sodium pertechnetate (Na^99m^TcO₄) contains technetium in its highest oxidation state ( + 7), it cannot directly form stable complexes with the nanoparticle formulation. Therefore, reduction of ^99m^Tc to a lower oxidation state is a prerequisite for efficient radiolabeling and stable complex formation with the ligand-associated nanoparticles [21]. Stannous chloride dihydrate (SnCl₂·2H₂O) was employed as the reducing agent owing to its well-established effectiveness in ^99m^Tc-radiopharmaceutical preparation [22].

To optimize the radiolabeling conditions, a systematic investigation of the critical formulation and reaction parameters affecting radiochemical yield was performed. Lyophilized CDX–CS-NPs were reconstituted in 0.01% HCl to obtain nanoparticle dispersions containing varying concentrations (100–500 µg/mL). Subsequently, 100 µL of freshly eluted Na^99m^TcO₄ solution (approximately 200 MBq) was added to each preparation. The amount of stannous chloride was varied between 50 and 150 µg to determine the optimal reducing-agent concentration required for efficient reduction of ^99m^Tc without promoting the formation of colloidal impurities. The reaction pH was adjusted and maintained at pH 4 based on preliminary optimization studies. The resulting mixtures were incubated at room temperature (25°C) for different time intervals (5, 15, 30, 45, and 60 min) to evaluate the effect of reaction time on labeling efficiency.

Formulation selection was based on achieving the highest radiochemical yield while maintaining the physicochemical integrity and stability of the nanoparticle system. Each parameter was evaluated individually while keeping the remaining variables constant, and the optimal formulation was identified as that providing maximum radiochemical yield with minimal levels of free pertechnetate and hydrolyzed-reduced technetium species. The optimized radiolabeling conditions were subsequently employed for the preparation of ^99m^Tc–CDX–CS-NPs and ^99m^Tc–CS-NPs used in all subsequent in vitro and in vivo investigations [23].

### Radiochemical yield of ^99m^Tc- CDX-CS-NPs

The radiochemical yield of ^99m^Tc–CDX–CS-NPs and ^99m^Tc–CS-NPs was determined using ascending paper chromatography. In this method, Whatman No. 1 chromatography paper strips (13 cm × 1 cm) were employed. A 10 µL aliquot of the reaction mixture was spotted 2 cm from the lower edge of each strip. The chromatograms were then developed in an ascending manner using acetone as the mobile phase for one strip, while a mixture of water: ethanol: ammonia (5:2:1, v/v/v) was used for the second strip [24]. After development, the strips were air-dried and subsequently cut into 1 cm segments. Each segment was individually analyzed for radioactivity using a NaI(Tl) gamma scintillation counter to determine the distribution of radioactivity along the chromatogram [25].

### In-vitro stability study

The in vitro stability of the ^99m^Tc–CDX–CS-NPs formulation was evaluated to assess its radiochemical integrity under physiological conditions. Briefly, 0.1 mL of the final radiolabeled preparation was incubated with freshly collected human serum at 37°C for 24h. At predetermined time intervals, the radiochemical purity was determined using the previously described paper chromatographic method [26], allowing monitoring of potential radiolysis over time.

### Murine models and in vivo studies

The in vivo evaluation of ^99m^Tc–CDX–CS-NPs and ^99m^Tc–CS-NPs was conducted using murine models divided into three experimental groups. The first group consisted of healthy mice that did not receive any exogenous infection and served as the control group. The second group was used to establish an infection model and received a clinical isolate of *Staphylococcus aureus*, obtained from the bacteriological culture collection of the Microbiology Unit at EAEA. The bacterial suspension, containing approximately 2 × 10⁸ CFU, was adjusted by dilution in sterile saline to obtain a turbid inoculum used for induction of septic foci. A 200 µL aliquot of the prepared suspension was injected into the left thigh muscle of each mouse, while the right thigh was left uninjected and used as an internal self-control. The third group was used to induce aseptic inflammation and received a 200 µL intramuscular injection of sterile turpentine oil into the left thigh, whereas the right thigh served as a control site. Each experimental group was further subdivided into four subgroups (n = 7 per subgroup). Animals in the infection and inflammation groups were monitored for 48–72 hours post-injection to confirm the development of visible inflammatory responses at the injection site, thereby ensuring successful model establishment [27–30].

For biodistribution studies, ^99m^Tc–CDX–CS nanoparticles (^99m^Tc–CDX–CS-NPs) and ^99m^Tc–CS-NPs were administered intravenously via the tail vein to all experimental groups at a dose of 3.7 MBq. Prior to in vivo application, the radiolabeled formulation was both purified and sterilized by passage through a 0.22 μm sterile membrane filter (Millipore, Bedford, MA, USA). This dual-purpose step ensured the efficient removal of unbound radionuclide species, potential aggregates, and microbial contaminants, thereby enhancing the radiochemical purity and overall safety of the formulation. The use of a 0.22 μm filter is particularly important for nanoparticulate systems, as it helps maintain a uniform particle size distribution while preventing embolic risk upon intravenous administration. Following this preparation, the formulation exhibited suitable physicochemical stability and sterility for systemic injection, allowing reliable evaluation of its in vivo biodistribution profile. Animals were maintained on a standard diet until sacrifice. Euthanasia was performed under tribromoethanol anesthesia (250–500 mg/kg, intraperitoneally), followed by cervical dislocation at predefined time points (0.5, 1-, 2-, and 3-hours post-injection) [31]. Subsequently, organs were excised, rinsed with saline, blotted dry, and weighed. The associated radioactivity was measured, and results were expressed as the percentage of injected dose per gram of tissue (%ID/g). Targeting efficiency was assessed by calculating the biodistribution ratios of the radiotracer between inflamed (septic and aseptic) and normal tissues (T/NT), as well as inflamed tissue to blood (T/B). These ratios were used to evaluate the selective accumulation of ^99m^Tc–CDX–CS-NPs and ^99m^Tc–CS-NPs at inflammatory sites, where higher uptake in inflamed tissue compared with blood and normal tissues is essential for improved imaging contrast, diagnostic accuracy, and reduced background interference.

### Statistical analysis

Statistical analysis was engaged using the student’s unpaired test. The statistical variances were considered to be acceptable when p < 0.05, and the results were detailed as mean ± SE (n = 7).

## Results and discussion

### Preparation and Characterization of nanoparticles

#### UV–VIS Analysis of CS-NPs, CDX, and CDX–CS-NPs

**Fig. 1:**
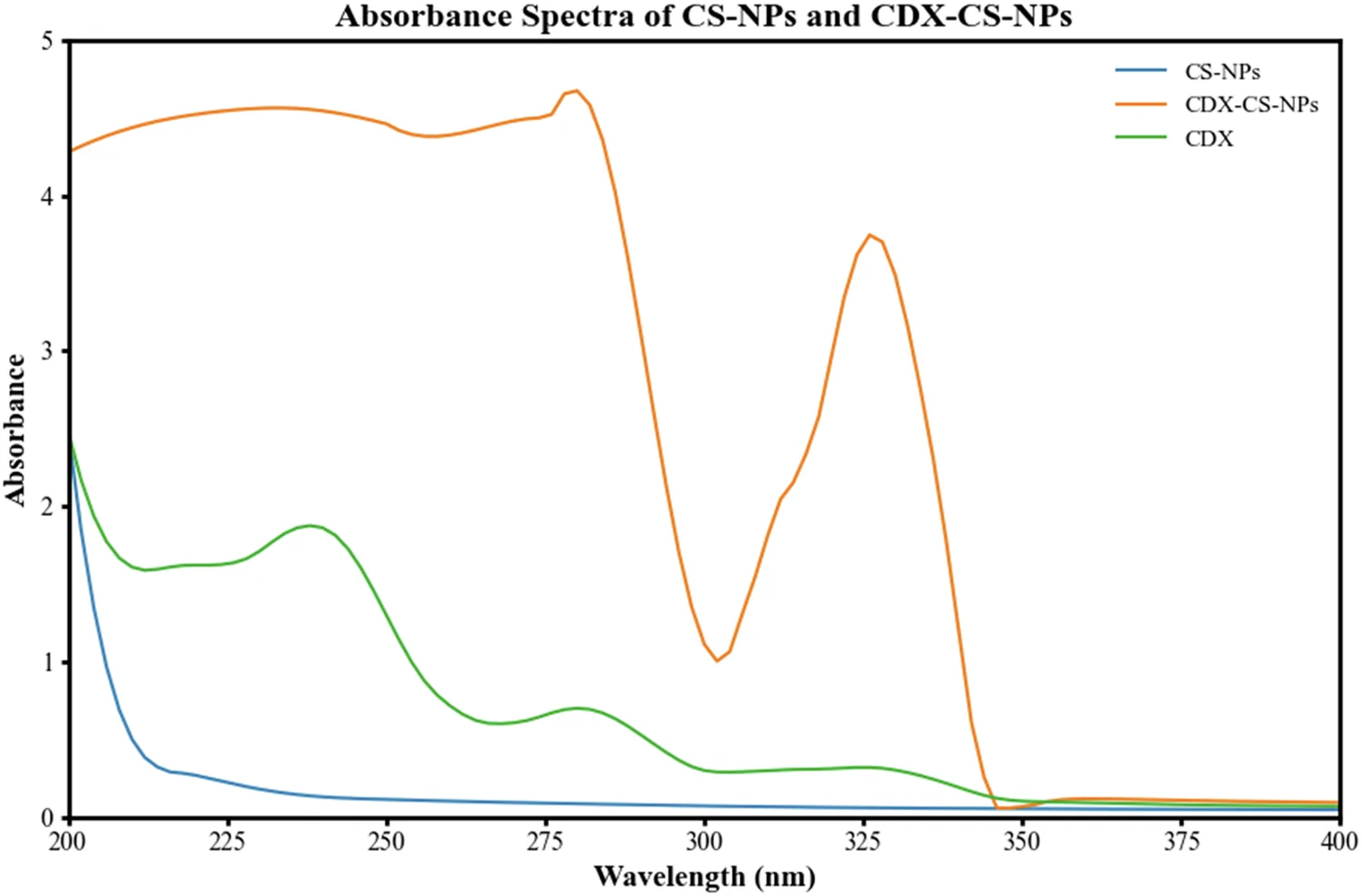
Absorbance Spectra of CS-NPs, CDX-CS-NPs and CDX

The UV–VIS absorption spectra of chitosan nanoparticles (CS-NPs), cefadroxil (CDX), and CDX-loaded CS-NPs (Fig. 1) demonstrate distinct optical characteristics that reflect their molecular structures and potential intermolecular interactions. CS-NPs exhibit a pronounced absorption band in the deep ultraviolet region (approximately 200–220 nm), followed by a steep decline in absorbance at higher wavelengths, which is consistent with the intrinsic electronic transitions of the chitosan backbone. In contrast, CDX displays a broader absorption profile extending from ~200 to 300 nm, with characteristic peaks observed around 230–240 nm and 270–280 nm, attributable to π → π* and n→π* transitions associated with its aromatic and carbonyl functional groups. Upon incorporation of CDX into the chitosan matrix, the resulting CDX–CS-NPs system shows notable spectral modifications, including an increase in absorbance intensity, peak broadening, and slight bathochromic shifts, particularly within the 270–280 nm region. Additionally, the emergence of a broad absorption band extending toward 320–330 nm is observed. These spectral changes provide strong evidence of significant drug–polymer interactions, most likely mediated by hydrogen bonding between the amino and hydroxyl groups of chitosan and the functional groups of CDX, confirming successful encapsulation and molecular-level association within the nanoparticulate system.

Encapsulation efficiency is the percentage of CDX loading content that can be entrapped into CS nanoparticles. The amount of drug with respect to the concentration of the CS nanoparticles had significant effect on the loading efficiency. The order of encapsulation efficiency was 90.02%, 97.63% and 91.03% for 25 mg, 50 mg and

100 mg CDX concentration taken for formulation using chitosan nanoparticles. The maximum of about 97.63% of entrapment efficiencywas found for 50 mg

#### XRD analysis

**Fig. 2:**
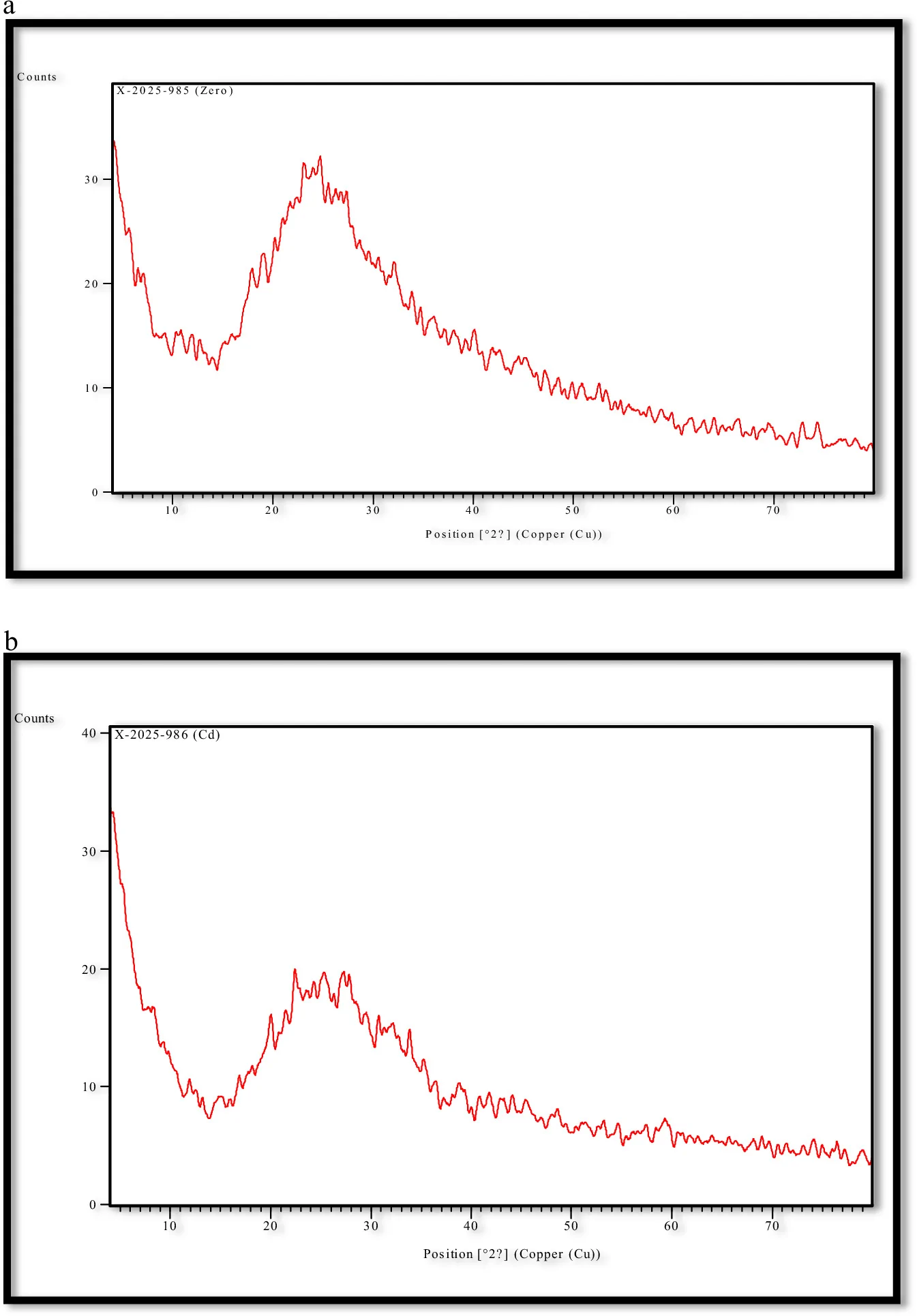
**a**. XRD pattern of Chitosan nanoparticles **b**. XRD pattern of CDX loaded Chitosan nanoparticles

X-ray diffraction (XRD) analysis was performed to distinguish the physical state of cefadroxil-loaded chitosan nanoparticles (CDX–CS-NPs) from that of the unloaded nanocarrier. The XRD pattern of blank chitosan nanoparticles (Fig. 2a) exhibited a broad diffraction peak at approximately 2θ = 22°, indicating their predominantly amorphous nature. In contrast, the CDX–CS-NPs formulation (Fig. 2b) showed two broad peaks at around 2θ = 22° and 28°, confirming the successful incorporation of cefadroxil within the chitosan matrix. The observed amorphous character can be attributed to ionic cross-linking between the functional groups of chitosan and sodium tripolyphosphate, as well as hydrogen bonding interactions between chitosan and cefadroxil. These intermolecular interactions likely disrupted the crystalline order of the individual components, leading to reduced crystallinity and enhanced molecular dispersion of the drug within the nanoparticle system, thereby indicating good compatibility and miscibility between cefadroxil and the chitosan-based carrier.

**Fig. 3:**
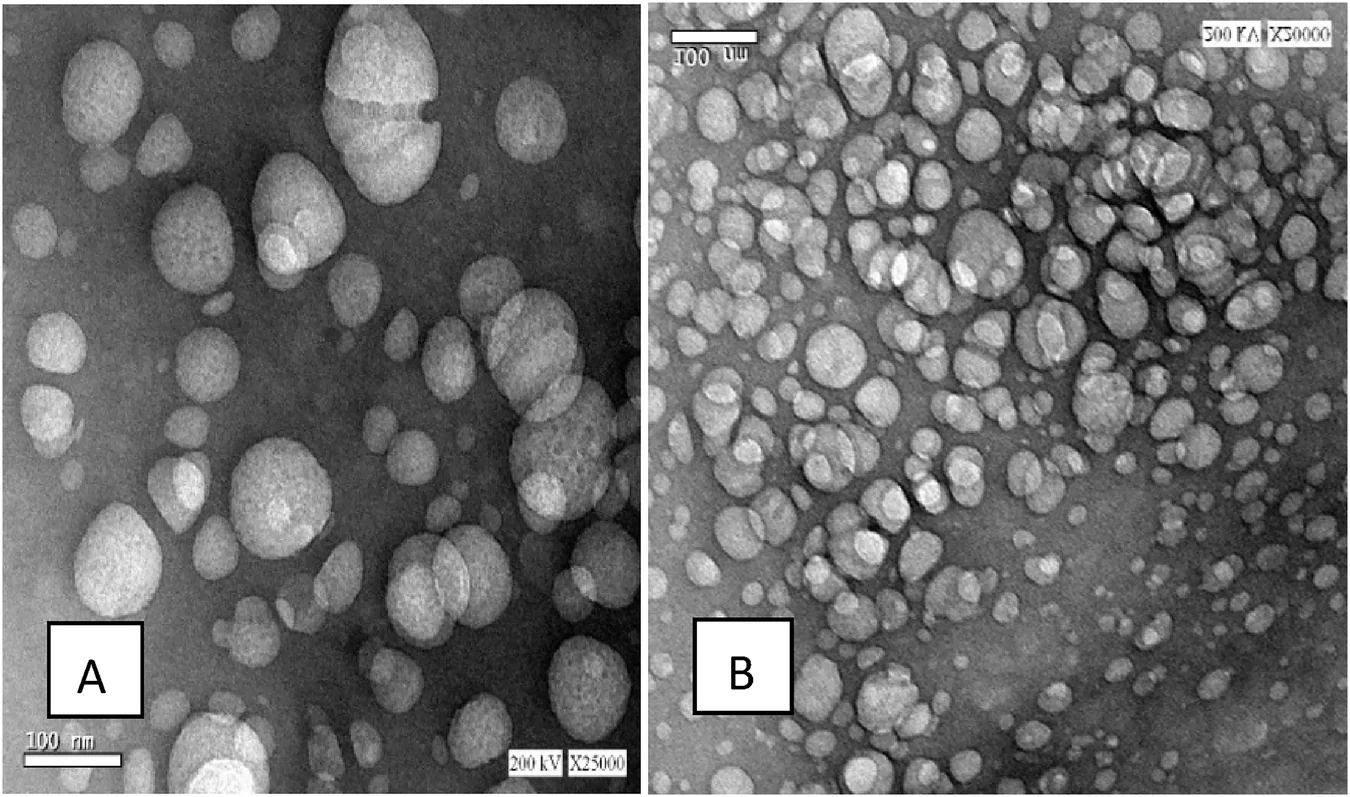
TEM image of: **a**. chitosan nanoparticles, **b**: CDX loaded chitosan nanoparticles

CDX-loaded chitosan nanoparticles were prepared using a simple ionotropic gelation method, with sodium tripolyphosphate (STPP) serving as the crosslinking agent. CDX, previously solubilized in acetone, was added dropwise to a homogeneous chitosan solution under continuous stirring. During this process, electrostatic interactions and hydrogen bonding between chitosan and CDX facilitate the entrapment of the drug within the polymeric matrix. Hydrogen bonds, which are short-range interactions, form only when donor and acceptor groups are in close proximity, resembling a stereocomplex-like arrangement. Upon the addition of STPP to the drug–polymer mixture, ionic crosslinking occurs through coacervation between oppositely charged species via noncovalent interactions [18]. Transmission electron microscopy (TEM) images (Fig. 3) demonstrate that the prepared nanoparticles possess a relatively uniform size distribution and a homogeneous, slightly rough surface morphology. The images also reveal clusters of predominantly spherical particles, consistent with the proposed structural model. Size measurements confirm the successful formation of nanoparticles, with diameters ranging from 30 to 80 nm for both unloaded and drug-loaded systems. Particle size analysis further indicates that these nanoparticles fall within the optimal size range for drug delivery applications, supporting their potential for effective tissue penetration and targeted delivery.

### Fourier transform infra-red (FT-IR) spectroscopy analysis

**Fig. 4:**
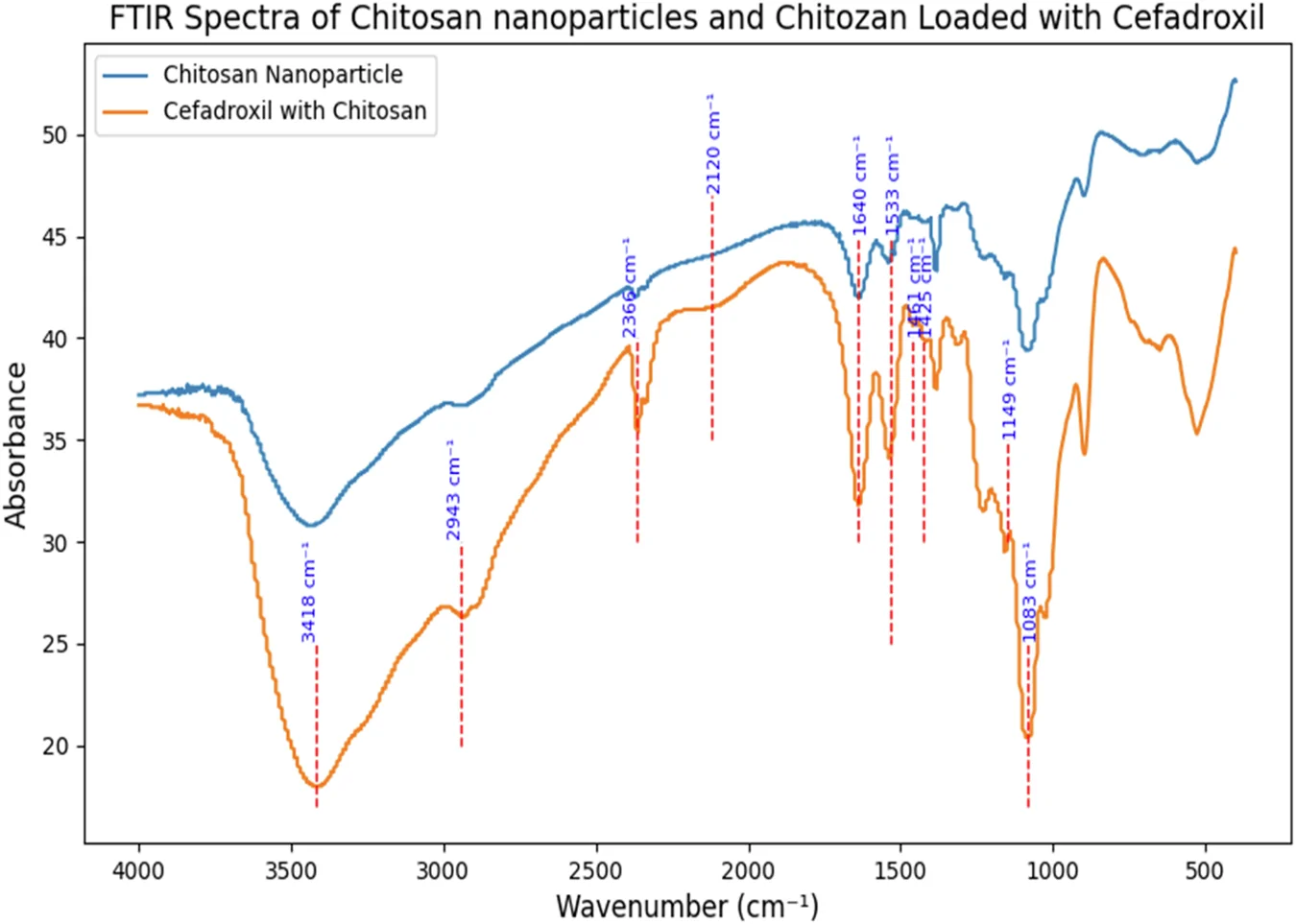
FTIR Spectrum of Chitosan nanoparticles and Chitosan loaded with Cefadroxil

Fourier-transform infrared (FT-IR) spectroscopy was utilized to investigate potential molecular interactions between chitosan nanoparticles and cefadroxil, as well as to confirm the successful incorporation of the drug within the polymeric matrix (Fig. 4). The FT-IR spectrum of pure chitosan exhibited its characteristic absorption bands. A broad and intense band in the 3400–3200 cm⁻¹ region corresponds to overlapping O–H and N–H stretching vibrations, indicative of extensive intra- and intermolecular hydrogen bonding within the chitosan structure [32]. Aliphatic C–H stretching vibrations were observed at approximately 2929 cm⁻¹. The characteristic amide bands of chitosan appeared at 1654 cm⁻¹ (Amide I, C = O stretching) and 1541 cm⁻¹ (Amide II, N–H bending). In addition, the polysaccharide backbone was confirmed by strong C–O–C stretching vibrations in the 1154–896 cm⁻¹ region, which are characteristic of the saccharide structure [33].

Upon comparison with the chitosan–cefadroxil formulation, clear spectral alterations were observed. The broad O–H/N–H stretching band shifted from approximately 3421 cm⁻¹ in pure chitosan to 3448 cm⁻¹ in the drug-loaded system, indicating the involvement of hydroxyl and amino groups of chitosan in hydrogen-bonding interactions with functional groups of cefadroxil [34]. The C–H stretching vibration observed near 2900 cm⁻¹ was still present in the formulation, although with reduced intensity, reflecting changes in the local molecular environment following drug incorporation. Moreover, pronounced shifts were detected in the amide region. The Amide I and Amide II bands of chitosan were shifted from 1654 to 1647 cm⁻¹ and from 1541 to 1530 cm⁻¹, respectively, in the chitosan–cefadroxil spectrum. These spectral changes are typically associated with hydrogen bonding and/or electrostatic interactions between the polymer matrix and drug molecules, thereby confirming the successful encapsulation of cefadroxil within the chitosan nanoparticle system [35].

In the polysaccharide fingerprint region (1200–900 cm⁻¹), the characteristic absorption bands of chitosan were retained; however, a noticeable increase in their intensity was observed following drug loading. This suggests minor conformational rearrangements within the chitosan backbone induced by interactions with the drug molecules, rather than any chemical degradation of the polymer structure [36]. In addition, a new absorption band appeared at 1773 cm⁻¹ in the chitosan–cefadroxil spectrum, which can be assigned to the carbonyl (C = O) stretching vibration of cefadroxil, thereby confirming its successful incorporation into the nanocomposite. Further evidence of molecular interactions between chitosan and the drug is provided by additional spectral changes observed in the 1500–1300 cm⁻¹ region, which support the presence of drug–polymer interactions within the system [37].

### Zeta Potential

**Fig. 5:**
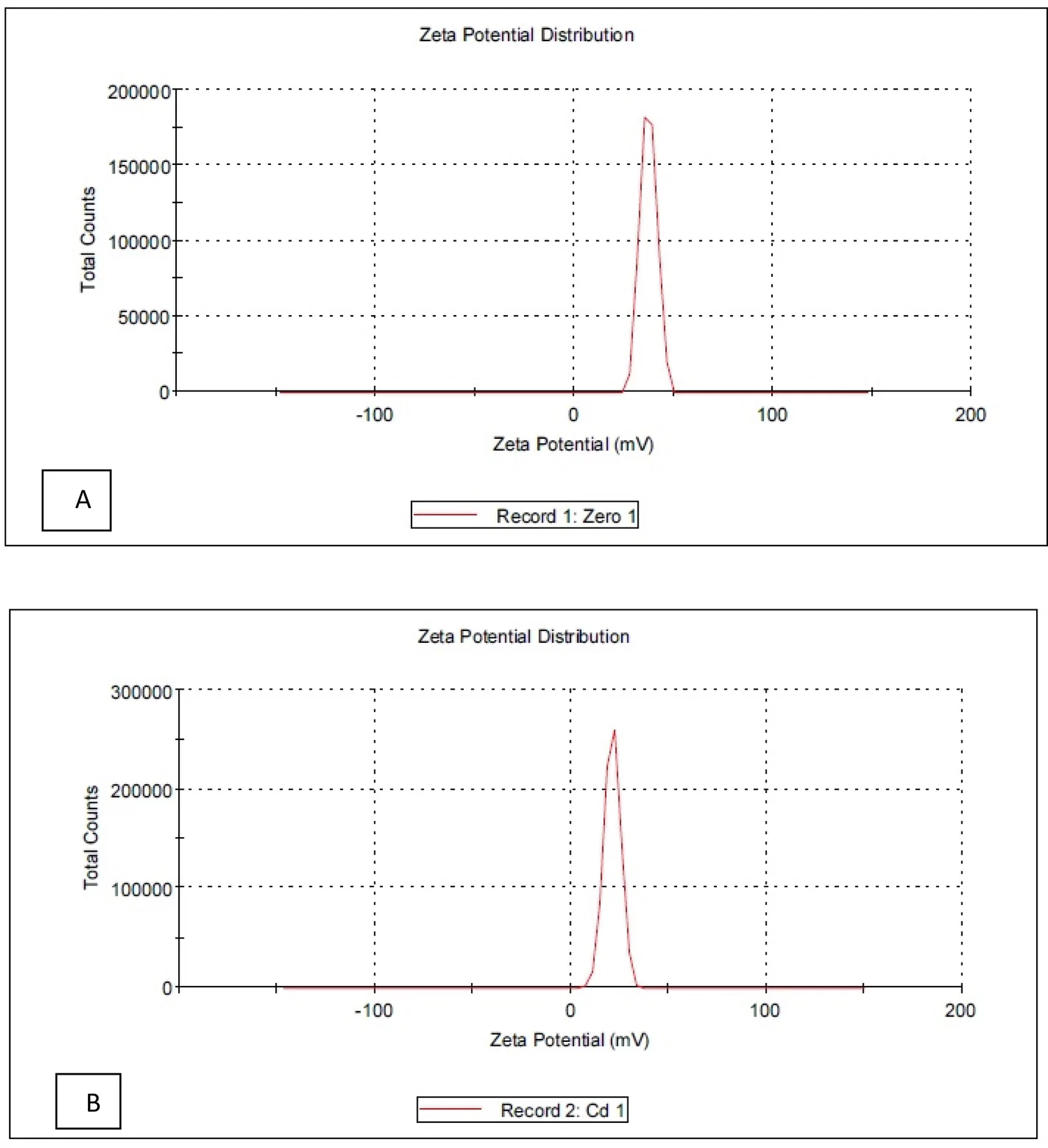
Zeta potential distribution of: **a**. chitosan nanoparticles, **b**: CDX loaded chitosan nanoparticles

Zeta potential analysis of the prepared chitosan nanoparticles (represented in Fig.5.A) is essential for evaluating their colloidal stability before and after drug loading. In this study, protonation of the amino groups of chitosan imparted a positive surface charge to the nanoparticles, as evidenced by a zeta potential value of +37.3 mV. Following drug loading (represented in fig. 5. B), this value decreased to +21.2 mV, which may be attributed to partial shielding of the positively charged surface by the encapsulated drug molecules. In addition, the presence of sodium tripolyphosphate (TPP) as an anionic cross-linker contributes to partial neutralization of chitosan’s protonated amino groups, thereby further reducing the overall surface charge [38] Although a reduction in zeta potential was observed after drug incorporation, the cefadroxil-loaded chitosan nanoparticles maintained acceptable colloidal stability. This stability can be explained by a combination of residual electrostatic repulsion and steric stabilization provided by the chitosan polymer matrix. The obtained stability was sufficient for the duration required to complete the study [39, 40].

### Radiolabeling of CDX-CS-NPs

**Fig. 6:**
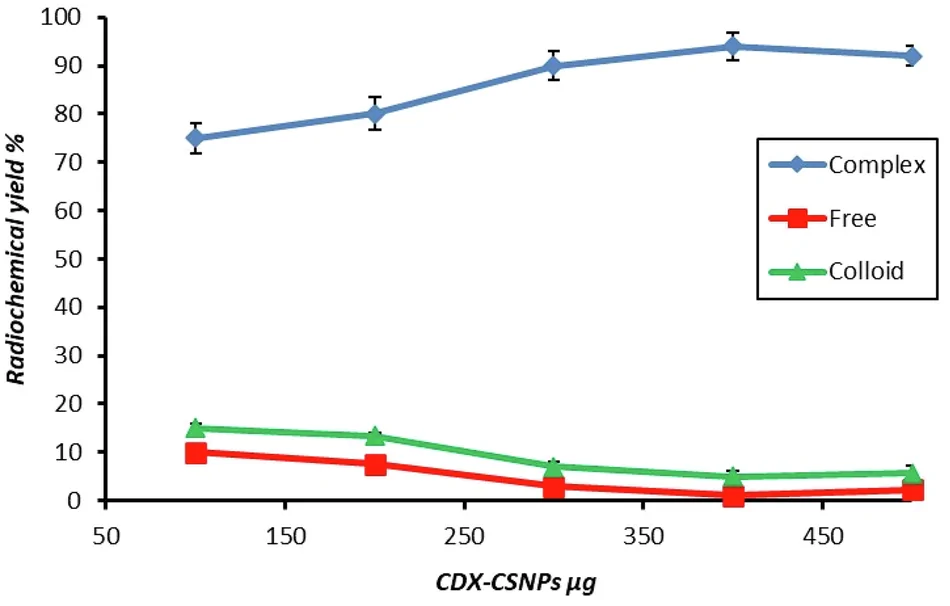
Radiochemical yield of ^99m^Tc-CDX-CS-NPscomplex as a function of CDX-CS-NPs

A simple, direct radiolabeling approach was employed to prepare ^99m^Tc–CDX–CS-NPs, achieving a maximum radiochemical yield (RY) of 94 ± 1.3% with stannous chloride dihydrate (SnCl₂·2H₂O) as the reducing agent. The role of the reducing agent is to convert ^99m^Tc from its +7-oxidation state to a lower oxidation state [21, 22], enabling its coordination with CDX–CS-NPs via a direct labeling method to obtain the desired radiopharmaceutical complex. This reduction step is critical for ensuring strong binding affinity and minimizing the formation of free or non-specifically bound radionuclide species. The effect of CDX–CS-NP concentration on RY is illustrated in Fig. 6, where concentrations ranging from 100 to 500 µg were evaluated. The results showed that RY increased progressively with increasing nanoparticle dose, reaching an optimal value of 94 ± 1.3% at 400 µg. However, a decline in RY was observed at higher concentrations, indicating that 400 µg is sufficient for efficient formation of ^99m^Tc–CDX–CS-NPs [41].

**Fig. 7:**
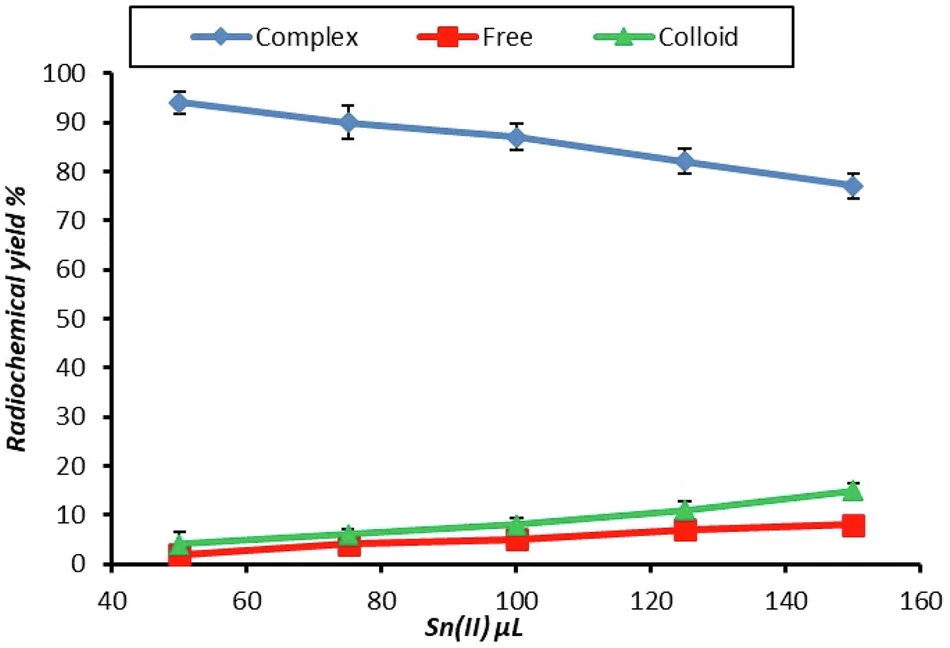
Radiochemical yield of ^99m^Tc- CDX-CS-NPs complex as a function of Tin chloride

Similarly, the amount of reducing agent was optimized by evaluating SnCl₂·2H₂O concentrations ranging from 50 to 150 µg (Fig. 7). A concentration of 50 µg was identified as optimal, providing sufficient reduction of ⁹⁹ᵐTcO₄⁻ to reactive lower-valent species while minimizing the risk of over-reduction. Excess SnCl₂ at higher concentrations can promote the formation of colloidal reduced hydrolyzed technetium species, thereby decreasing labeling efficiency. Under optimal conditions, an RY of 94 ± 1.3% was achieved, confirming efficient radiocomplex formation.

From a formulation standpoint, chitosan nanoparticles offer a multifunctional platform that extends beyond conventional radiolabeled drug systems such as ⁹⁹ᵐTc–CDX alone, providing enhanced stability, improved targeting efficiency, and potential synergistic therapeutic properties. Compared with hyaluronic acid-based nanoparticles, chitosan exhibits broader, receptor-independent accumulation in inflamed tissues, as well as stronger radiolabeling capacity and superior mucoadhesive and electrostatic interactions. These properties make chitosan particularly advantageous for infection- and inflammation-imaging applications [42, 43].

**Fig. 8:**
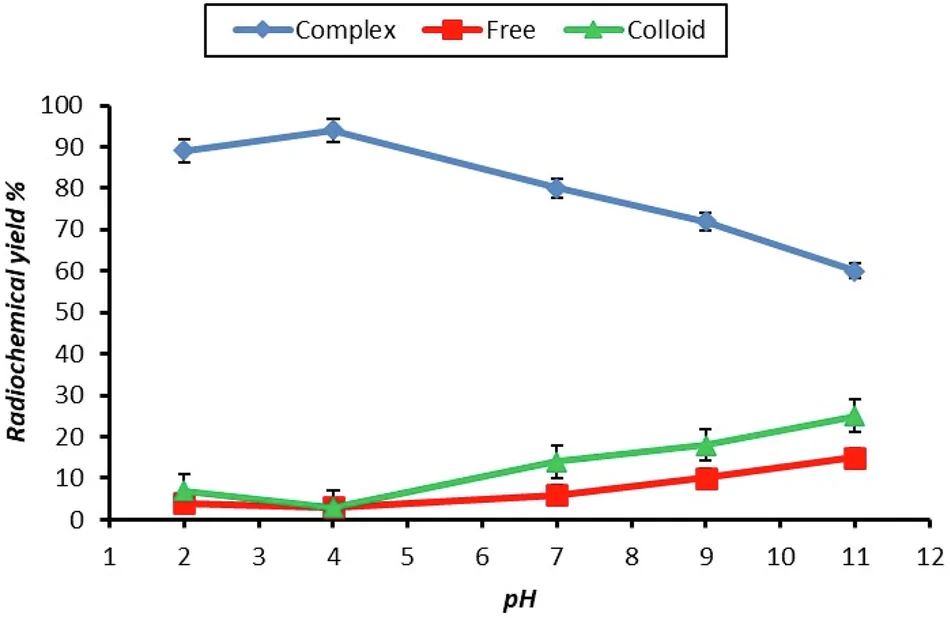
Radiochemical yield of ^99m^Tc- CDX-CS-NPs complex as a function of pH medium

Given that chitosan is highly pH-sensitive due to protonation of its amino groups under acidic conditions [44], the effect of pH on radiolabeling efficiency was systematically investigated. As shown in Fig. 8, pH 4 was identified as the optimal condition, yielding the highest RY of 94 ± 1.3%. Under acidic conditions, increased protonation enhances chitosan interactions with technetium species, thereby improving complex stability. In contrast, at neutral and alkaline pH values, a significant reduction in labeling efficiency was observed, accompanied by increased colloid formation. This behavior may be attributed to the precipitation of SnCl₂ under alkaline conditions and the enhanced formation of reduced hydrolyzed ⁹⁹ᵐTc colloids, both of which compete with the desired labeled complex formation [45, 46].

**Fig. 9:**
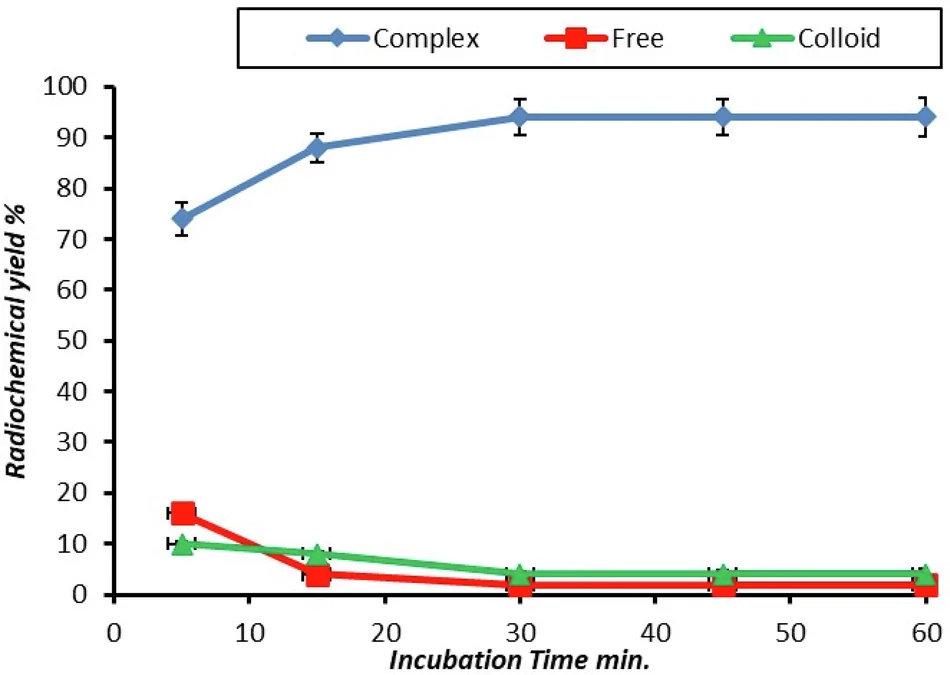
Radiochemical yield of ^99m^Tc- CDX-CS-NPs complex as a function of incubation time

Furthermore, the effect of incubation time on the RY of ^99m^Tc–CDX–CS-NPs was evaluated, as shown in Fig. 9. The complexation process between CDX–CS-NPs and ^99m^Tc ions occurred rapidly, with an initial RY of 74 ± 1.1% observed after 5 min at room temperature. The RY increased with time, reaching a maximum of 94 ± 1.3% after 30 min of incubation. Beyond this time point, no further significant change in RY was observed, indicating that equilibrium had been reached [47]. These results collectively demonstrate that the optimized conditions ensure rapid, efficient, and stable radiolabeling suitable for subsequent biological applications.

### Radiolabeling of chitosan nanoparticles

Radiolabeling of chitosan nanoparticles (100 μg dispersed in sodium acetate solution, 1 mg/mL) with technetium-99m was successfully optimized under various reaction conditions. The highest radiolabeling efficiency was achieved at pH 4 using 50 μg of stannous chloride (SnCl₂) as the reducing agent, with an incubation period of 30 min. Under these optimized conditions, the radiochemical yield reached 90.0 ± 0.5%, indicating efficient reduction of pertechnetate and stable incorporation of ^99m^Tc into the chitosan nanoparticle matrix. The low standard deviation observed among replicate experiments demonstrates the excellent reproducibility and reliability of the radiolabeling procedure. These findings suggest that the selected reaction parameters provide favorable conditions for achieving high radiochemical purity, thereby supporting the suitability of the radiolabeled chitosan nanoparticles for subsequent biological evaluation and in vivo imaging applications.

### The in-vitro stability of ^99m^Tc- CDX-CS-NPs

**Fig. 10:**
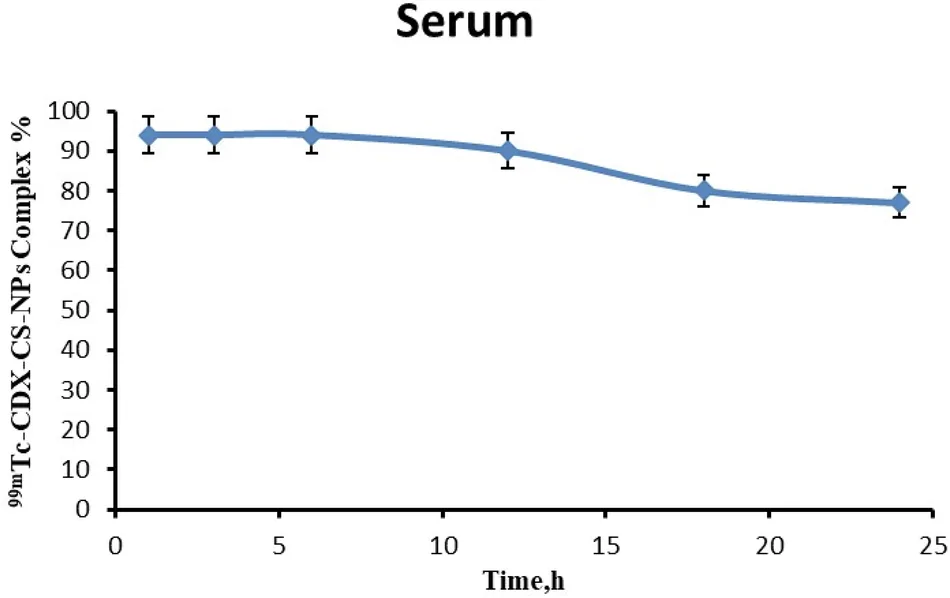
In Vitro Stability of ^99m^Tc- CDX-CS-NPs in fresh serum at various time intervals post radiolabeling

Determining in vitro stability is a critical step in evaluating radiolabeled compounds prior to in vivo application. It provides essential information about the integrity of the radiopharmaceutical under physiological conditions and predicts its behavior after administration. A radiolabeled compound must maintain its structural and radiochemical stability in biological media, such as serum, to ensure that the radioactive label remains bound to the intended carrier without premature dissociation. Loss of stability in vitro may lead to the release of free radionuclide, which can result in non-specific distribution, increased background signal, and accumulation in non-target organs. This not only reduces imaging contrast and diagnostic accuracy but may also increase radiation dose to healthy tissues. Therefore, in vitro stability studies help to assess the resistance of the radiocomplex to processes such as trans-chelation, radiolysis, and protein binding in serum [48]. As illustrated in Fig. 10, the radiochemical yield (RY) of ^99m^Tc–CDX–CS-NPs remained stable at 94 ± 1.3% for up to 6 hours post-radiolabeling, indicating good in vitro stability in serum. Over time, a gradual decline in radiochemical purity was observed, reaching 77 ± 1.4% after 24 hours. Based on these findings, ^99m^Tc–CDX–CS-NPs can be considered a reliable radiotracer for diagnostic applications within approximately 6 hours following preparation.

### Entrapment efficiency

Encapsulation efficiency (EE%) represents the proportion of cefadroxil (CDX) successfully entrapped within the chitosan (CS) nanoparticles relative to the initial amount used in the formulation. In the present study, an encapsulation efficiency of 97.6% was achieved when 5 mg of CDX was incorporated into the chitosan nanoparticle system, indicating a highly efficient drug-loading capacity.

### In-vivo studies for ^99m^Tc- CDX-CS-NPs

The biological evaluation of ⁹⁹ᵐTc–CDX–CS–NPs is essential to assess their suitability as a radiotracer for detecting inflammatory foci. Initially, biodistribution studies were conducted in normal mice to investigate pharmacokinetics and confirm that the radiolabeling process did not adversely affect the biological properties of the CDX–CS nanoparticulate system. As summarized in Table 1, the radiotracer exhibited an initial blood uptake of 10.9 ± 1.3% at 30 min post-injection, followed by a rapid decline to 1.1 ± 0.1% at 3 h. This decrease is likely attributed to limited plasma protein binding [49, 50], indicating rapid systemic distribution and minimal long-term circulation. Such a profile is advantageous because it reduces systemic toxicity and nonspecific tissue accumulation. Furthermore, ⁹⁹ᵐTc–CDX–CS–NPs demonstrated minimal uptake by the reticuloendothelial system (RES), as evidenced by the low levels of radioactivity detected in the spleen, lungs, and bones across all time points. Renal excretion was identified as the primary elimination pathway, with urinary activity reaching 33 ± 2.1% at 3 h post-injection [51, 52]. Intestinal uptake of the radiolabeled formulation was initially low (1.98 ± 0.2% shortly after administration) but increased progressively over time, reaching 8.3 ± 0.9% at 3 h post-injection. This time-dependent accumulation suggests sustained interaction of the nanoparticles with the intestinal epithelium. Such behavior can be partly attributed to cefadroxil, which is efficiently absorbed via the human peptide transporter 1 (hPEPT1), facilitating trans-epithelial transport [53, 54]. In addition, chitosan-based nanoparticles enhance intestinal retention through their mucoadhesive properties, prolonged residence time, and ability to transiently open tight junctions, thereby promoting paracellular transport and improving overall drug absorption [55, 56].

**Table 1:** Biodistribution of ^99m^Tc- CDX-CS-NPs Tracer in various organs and body fluids in Normal mice versus different time intervals (%ID/g tissue ± S.E, n = 7)

Organs &Body Fluid	^99m^Tc- CDX-CS-NPs % ID/g tissue			
	Time post-injection (h.)			
	0.5 h.	1 h.	2 h.	3 h.
Blood	10.9 ± 1.3	4.2 ± 0.5	2.3 ± 0.2	1.1 ± 0.1
Bone	0.4 ± 0.05	0.3 ± 0.01	0.2 ± 0.01	0.1 ± 0.01
Muscles	0.7 ± 0.02	0.55 ± 0.01	0.42 ± 0.02	0.21 ± 0.01
Brain	0.4 ± 0.01	0.2 ± 0.01	0.1 ± 0.001	0.02 ± 0.001
Stomach	2.1 ± 0.1	1.79 ± 0.15	1.4 ± 0.1	0.6 ± 0.02
Intestine	1.98 ± 0.2	3.5 ± 0.3	5.7 ± 0.3	8.3 ± 0.9
Kidney	3.2 ± 0.2	6.8 ± 0.3	8.3 ± 0.2	3.2 ± 0.2
Liver	2.8 ± 0.5	4.4 ± 0.3	1.9 ± 0.1	1.1 ± 0.1
Spleen	0.7 ± 0.01	1.3 ± 0.1	0.6 ± 0.01	0.38 ± 0.01
Lung	2.2 ± 0.2	1.5 ± 0.1	1.3 ± 0.1	1 ± 0.01
Heart	1.9 ± 0.2	1.5 ± 0.2	1.3 ± 0.1	0.9 ± 0.01
Urine	3.7 ± 0.6	14.3 ± 1.1	26 ± 1.9	33 ± 2.1

Additionally, the low levels of radioactivity detected in the stomach over time indicate the high *in vivo* stability of ⁹⁹ᵐTc–CDX–CS–NPs. Brain uptake was minimal, reaching only 0.4 ± 0.01% at 30 min post-injection, likely due to the formulation’s limited ability to cross the blood–brain barrier [57]. Moderate pulmonary uptake was observed, with lung accumulation of 2.2 ± 0.2% at 30 min post-injection, suggesting effective interaction with lung tissue and pleural fluid [58]. In contrast, muscle uptake was relatively low (0.7 ± 0.02% at 30 min) and decreased further to 0.21 ± 0.01% at 3 h post-injection, indicating minimal non-specific distribution to peripheral tissues. The majority of the administered dose of ⁹⁹ᵐTc–CDX–CS–NPs was rapidly metabolized and eliminated via renal excretion, consistent with a short biological half-life of approximately 2 h following intravenous administration [59–61]. This rapid clearance profile is advantageous, as it reduces radiation exposure and enhances the radiotracer’s safety for potential clinical applications. One-way ANOVA revealed statistically significant differences in radiotracer uptake among the evaluated time points (P < 0.05) in all examined organs, demonstrating a pronounced time-dependent biodistribution pattern. The significant decline in radioactivity levels over time indicates efficient clearance of the tracer from the systemic circulation and non-target tissues. Quantitative analysis further showed that tracer elimination occurred predominantly through the renal pathway, as evidenced by the characteristic uptake profile in the kidneys and urinary bladder. The statistically significant temporal changes observed across organs suggest rapid pharmacokinetic redistribution and excretion of the radiotracer, resulting in progressively lower retention within normal tissues. Such findings are indicative of favorable biological behavior, as limited long-term accumulation in non-target organs can reduce background activity and improve target-to-background contrast for imaging applications. Collectively, the ANOVA results confirm that the observed variations in organ uptake are attributable to true time-dependent biological processes rather than random experimental variation, supporting the conclusion that the tracer exhibits rapid blood clearance, predominant renal excretion, and minimal prolonged retention in healthy tissues.

Inflammatory sites are characterized by increased vascular permeability, immune cell recruitment, and elevated metabolic activity, all of which promote the preferential accumulation of nanoparticles at these regions. When antibiotics are incorporated into nanoparticulate carriers and radiolabeled, they can selectively localize to infection-associated inflammatory foci, thereby enhancing imaging contrast and improving diagnostic accuracy in nuclear medicine [62]. In addition to their carrier function, chitosan nanoparticles may contribute intrinsically to antibacterial activity, which is strongly influenced by chitosan’s molecular weight (Mw). In the present study, a medium-molecular-weight chitosan was employed, as lower-molecular-weight fractions have been reported to exhibit superior antibacterial efficacy. Notably, a marked enhancement in activity against *S. aureus* has been observed when the molecular weight of chitosan is reduced c and sterile inflammatory procesto below 300 kDa [63, 64]. This improvement is primarily attributed to increased solubility and greater chain mobility, which facilitate stronger electrostatic interactions between the positively charged amino groups of chitosan and the negatively charged bacterial cell membrane. Such interactions can compromise membrane integrity, leading to increased permeability, leakage of intracellular components, and ultimately bacterial cell death. Therefore, optimizing chitosan’s molecular weight is critical to maximizing both its antimicrobial performance and its effectiveness as a drug-delivery platform. In this context, the selected chitosan system not only enhances nanoparticle stability and targeting but may also synergistically improve the overall therapeutic and diagnostic performance of the radiolabeled formulation.

The administration of the ^99m^Tc-CDX-CS-NPs tracer in S. aureus-inflamed mice is presented in Table 2. The radiotracer exhibited significant accumulation in the infected (septic) muscle, with an uptake of 3.34 ± 0.05% at 30 min post-injection, increasing to a maximum of 6.16 ± 0.6% at 2 h post-injection. This progressive increase reflects both passive targeting mechanisms, such as the enhanced permeability and retention (EPR) effect, and active biological interactions. In particular, the bactericidal activity of cefadroxil against S. aureus likely contributes to enhanced retention through binding to bacterial cell wall components and interaction with actively proliferating microorganisms [65, 66]. Additionally, the chitosan matrix may further promote localization through its mucoadhesive properties and electrostatic interactions with negatively charged bacterial surfaces and inflamed tissues. In contrast, uptake in the non-inflamed muscle (control) was markedly lower, measuring 0.94 ± 0.03% at 30 min and decreasing to 0.55 ± 0.02% at 3 h post-injection. Statistical analysis using Student’s unpaired t-test (P < 0.05) confirmed that the difference in radiotracer accumulation between infected and normal muscle tissues was significant at all evaluated time points. This finding demonstrates the ability of the radiolabeled formulation to effectively distinguish infected tissue from healthy tissue and highlights its potential utility as an infection-imaging agent. The consistently higher uptake observed in infected muscle suggests that the tracer possesses favorable targeting characteristics and exhibits selective retention within sites of infection. The target-to-non-target (T/NT) ratio was calculated to evaluate the ability of ⁹⁹ᵐTc–CDX–CS–NPs to selectively localize at infected foci. As shown in Table 2, the T/NT ratio reached a maximum value of 8.55 at 2 h post-injection, indicating high specificity for infection imaging. Furthermore, the target-to-blood (T/B) ratio was assessed to determine the optimal imaging time. The T/B ratio reached peak values of 4.02 and 4.88 at 2 and 3 h post-injection, respectively, suggesting that this time window provides optimal contrast for accurate diagnosis and potential therapeutic planning. This trend reflects rapid systemic clearance combined with sustained retention at the infection site, resulting in improved contrast.

**Table 2:** Biodistribution of ^99m^Tc- CDX-CS-NPs Tracer in various organs and body fluids in mice bearing staphylococcus aureus versus different time intervals (%ID/g tissue ± S.E, n = 7)

Organs &Body Fluid	^99m^Tc- CDX-CS-NPs % ID/g tissue Infected mice with S. aureus			
	Time post-injection (h.)			
	0.5 h.	1 h.	2 h.	3 h.
Blood	11.5 ± 0.2	3.87 ± 0.2	1.53 ± 0.2	0.86 ± 0.1
Bone	0.57 ± 0.1	0.47 ± 0.02	0.34 ± 0.01	0.27 ± 0.01
**Muscles (NT)**	**0.94** ± **0.03**	**0.84** ± **0.01**	**0.72** ± **0.03**	**0.55** ± **0.02**
**Muscles (T)**	**3.34** ± **0.05**	**5.3** ± **0.1**	**6.16** ± **0.6**	**4.2** ± **0.5**
Brain	0.42 ± 0.01	0.16 ± 0.01	0.06 ± 0.0	0.02 ± 0.0
Stomach	1.85 ± 0.02	1.74 ± 0.1	1.33 ± 0.1	0.93 ± 0.03
Intestine	1.8 ± 0.03	3.1 ± 0.1	5.1 ± 0.2	7.7 ± 0.8
Kidney	4.6 ± 0.2	6.4 ± 0.2	8.87 ± 0.8	2.27 ± 0.3
Liver	2.1 ± 0.1	3.87 ± 0.2	1.1 ± 0.1	0.75 ± 0.1
Spleen	0.63 ± 0.02	1.1 ± 0.05	0.51 ± 0.06	0.32 ± 0.03
Lung	2.5 ± 0.01	1.2 ± 0.03	1.18 ± 0.2	0.86 ± 0.02
Heart	2.1 ± 0.2	1.16 ± 0.02	0.95 ± 0.01	0.72 ± 0.01
Urine	3.6 ± 0.2	10.5 ± 0.9	28.5 ± 1.3	37 ± 1.8
Stool	-	-	8.12 ± 1.1	-
**T/NT**	**3.55**	**6.3**	**8.55**	**7.63**
**T/B**	**0.29**	**1.36**	**4.02**	**4.88**

In addition, the *in vivo* behavior of ⁹⁹ᵐTc–CDX–CS–NPs was evaluated in murine models bearing sterile inflammation induced by turpentine oil, as presented in Table 3. The radiotracer demonstrated appreciable retention at the inflamed site, with uptake peaking at 2.65 ± 0.2% at 2 h post-injection, followed by a gradual washout to 1.82 ± 0.04% at 3 h. This transient retention profile may be attributed to the absence of a microbial target in sterile inflammation, where localization is primarily governed by enhanced vascular permeability and passive diffusion rather than specific antibiotic–bacteria interactions. In contrast, uptake in the normal muscle (control) was significantly lower, measuring 0.90 ± 0.01% at 30 min post-injection and decreasing to 0.36 ± 0.02% at 3 h post-injection. Statistical analysis using Student’s unpaired t-test demonstrated that radiotracer accumulation in inflamed muscle was significantly higher than that observed in normal muscle tissue at all evaluated time points (P < 0.05). The persistence of statistically significant differences throughout the study indicates that the observed increase in tracer uptake was unlikely to have occurred by chance and reflects a true biological distinction between inflamed and healthy tissues. These findings suggest that the radiotracer exhibits a consistent and reproducible affinity for sites of inflammation, resulting in preferential localization within pathological tissues. The target-to-non-target (T/NT) ratio reached a maximum value of 6.02 at 2 h post-injection, indicating that ⁹⁹ᵐTc–CDX–CS–NPs can effectively localize in aseptic inflammatory foci. However, this value is lower than that typically observed in infection models, further supporting the contribution of specific antibiotic–bacterial interactions in enhancing tracer retention. Furthermore, the target-to-blood (T/B) ratio peaked at 2.84 at 3 h post-injection, suggesting that this time point is optimal for imaging to achieve improved contrast and diagnostic clarity. This trend reflects progressive blood clearance combined with sustained, albeit declining, retention at the inflamed site.

**Table 3:** Biodistribution of ^99m^Tc- CDX-CS-NPs Tracer in various organs and body fluids in inflamed mice with turpentine oil versus different time intervals (%ID/g tissue ± S.E, n = 7)

Organs &Body Fluid	^99m^Tc- CDX-CS-NPs % ID/g tissue Inflamed mice with turpentine oil			
	Time post-injection (h.)			
	0.5 h.	1 h.	2 h.	3 h.
Blood	9.2 ± 0.4	4.1 ± 0.1	1.75 ± 0.2	0.64 ± 0.1
Bone	0.52 ± 0.01	0.45 ± 0.02	0.30 ± 0.02	0.12 ± 0.03
**Muscles (NT)**	**0.90** ± **0.01**	**0.63** ± **0.03**	**0.44** ± **0.03**	**0.36** ± **0.02**
**Muscles (T)**	**2.13** ± **0.1**	**2.58** ± **0.2**	**2.65** ± **0.2**	**1.82** ± **0.04**
Brain	0.33 ± 0.02	0.19 ± 0.01	0.09 ± 0.01	0.03 ± 0.0
Stomach	1.96 ± 0.2	1.36 ± 0.1	0.97 ± 0.0.4	0.42 ± 0.02
Intestine	1.48 ± 0.15	4.23 ± 0.2	5.8 ± 0.6	6.5 ± 0.2
Kidney	2.89 ± 0.3	6.32 ± 0.3	7.82 ± 1.1	1.95 ± 0.1
Liver	2.38 ± 0.2	3.98 ± 0.2	1.5 ± 0.3	0.62 ± 0.03
Spleen	1.12 ± 0.1	1.58 ± 0.2	0.42 ± 0.02	0.18 ± 0.01
Lung	2.13 ± 0.2	1.78 ± 0.1	0.62 ± 0.01	0.38 ± 0.01
Heart	1.96 ± 0.2	1.43 ± 0.12	0.85 ± 0.02	0.68 ± 0.01
Urine	2.7 ± 0.3	12.5 ± 0.8	23 ± 1.5	31.5 ± 1.5
Stool	-	-	4.3 ± 0.4	-
**T/NT**	**2.36**	**4.09**	**6.02**	**4.47**
**T/B**	**0.23**	**0.62**	**1.5**	**2.84**

The results demonstrated a numerical increase in the measured parameter in septic mice compared with aseptic mice, corresponding to an approximately 54% higher mean value. However, statistical analysis indicated that this difference did not reach significance (p = 0.137). The absence of statistical significance may be attributed, at least in part, to the variability observed within each experimental group, which can reduce the ability of the statistical test to detect true differences between groups. Despite the lack of statistical significance, the observed trend toward higher uptake in septic mice is noteworthy and may reflect underlying biological differences between septic and sterile inflammatory processes.

**Fig. 11:**
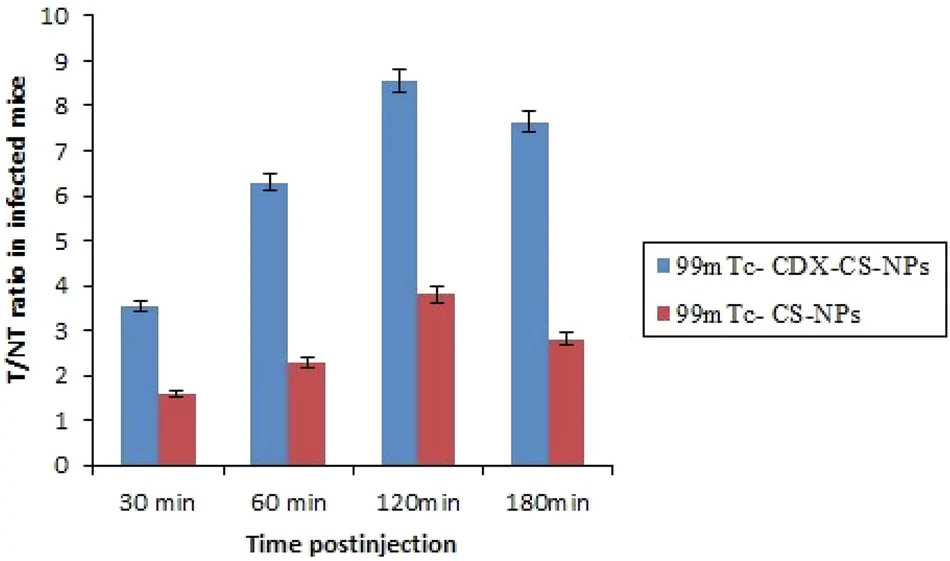
Comparative assessment of infection localization by ⁹⁹ᵐTc–CDX–CS–NPs and ⁹⁹ᵐTc–CS–NPs using Target-to-Non-Target (T/NT) ratios in infected mice

Following intravenous administration of ⁹⁹ᵐTc–CS–NPs to infected mice, the radiotracer exhibited predominant accumulation in the liver, indicating substantial uptake by the reticuloendothelial system (RES), which is consistent with the known biodistribution profile of chitosan-based nanoparticles [67]. In contrast, relatively low levels of radioactivity were detected in most other organs, suggesting limited nonspecific distribution throughout the body. Evaluation of the target-to-non-target (T/NT) ratio revealed only modest localization of ⁹⁹ᵐTc–CS–NPs at the site of infection. The T/NT ratio gradually increased with time and reached a maximum value of 3.8 at 2 h post-injection (Fig. 11), indicating a moderate ability of the radiolabeled chitosan nanoparticles to discriminate infected tissue from surrounding normal tissue.

When compared with the corresponding biodistribution profile of ⁹⁹ᵐTc–CDX–CS–NPs, a markedly lower accumulation of ⁹⁹ᵐTc–CS–NPs was observed within the infected lesions. This finding suggests that the enhanced localization of ⁹⁹ᵐTc–CDX–CS–NPs at sites of infection is primarily attributable to the incorporation of cefadroxil within the chitosan nanoparticle matrix. The presence of cefadroxil likely promotes preferential interaction with bacterial cells and infected tissues, thereby improving target specificity and increasing radiotracer retention at the infection focus. Consequently, the antibiotic-loaded formulation demonstrated superior infection-targeting capability compared with the unloaded chitosan nanoparticles.

Furthermore, statistical analysis using Student’s unpaired t-test confirmed that the uptake of ⁹⁹ᵐTc–CDX–CS–NPs in infected lesions was significantly higher than that of ⁹⁹ᵐTc–CS–NPs at all investigated time points (P < 0.05). These results demonstrate that cefadroxil loading significantly enhances the infection-targeting efficiency of chitosan nanoparticles and supports the potential application of ⁹⁹ᵐTc–CDX–CS–NPs as a more sensitive and specific radiotracer for the detection of bacterial infections.

Overall, these findings suggest that ⁹⁹ᵐTc–CDX–CS–NPs can accumulate in sterile inflammatory lesions via passive targeting mechanisms, including the enhanced permeability and retention (EPR) effect and the mucoadhesive and permeation-enhancing properties of chitosan. The optimal imaging window appears to be between 2 and 3 h post-injection, where a balance between target uptake and background clearance is achieved, thereby improving image contrast and diagnostic accuracy. All inclusive, these findings validate that ⁹⁹ᵐTc–CDX–CS–NPs possess favorable targeting characteristics for infection imaging. The optimal imaging window seems to lie between 2 and 3 h post-injection, where maximal target uptake accords with reduced background activity, thereby enabling high-contrast visualization and supporting precise diagnosis and potential therapeutic decision-making.

Compared with conventional radiolabeled antibiotics, the developed ⁹⁹ᵐTc–CDX–CS–NP system demonstrates markedly improved performance. For example, ⁹⁹ᵐTc-labeled ceftriaxone shows a gradual increase in the target-to-non-target (T/NT) ratio, reaching a maximum of only 4.5 at 24 h post-injection, indicative of slower accumulation and limited target specificity [68]. Similarly, other radiolabeled antibiotics, such as ⁹⁹ᵐTc–ciprofloxacin [69] and ⁹⁹ᵐTc–levofloxacin [70], typically exhibit moderate T/NT ratios in the range of 2.0–5.0 over comparable or longer time intervals. This relatively modest performance is often attributed to nonspecific distribution, rapid diffusion away from the target site, and suboptimal pharmacokinetic profiles, which can reduce imaging contrast and delay optimal imaging time points. In contrast, nanoparticle-based radiopharmaceuticals have consistently demonstrated enhanced targeting efficiency and improved imaging characteristics. For instance, radiolabeled vancomycin-loaded nanoparticles [71] and liposomal antibiotic systems [72] have been reported to achieve T/NT ratios exceeding 5.0 within a few hours post-injection. This superior performance is largely attributed to the unique physicochemical properties of nanoparticulate carriers, including improved in vivo stability, prolonged circulation time, and preferential accumulation at infection sites through the enhanced permeability and retention (EPR) effect. Moreover, nanoparticle systems can facilitate higher local drug concentrations and stronger interactions with bacterial components, thereby enhancing retention at the target site.

Within this context, the high T/NT ratios achieved by ⁹⁹ᵐTc–CDX–CS–NPs, particularly within 2–3 h post-injection, highlight their rapid targeting capability and superior specificity compared to conventional radiolabeled antibiotics. These findings underscore the potential of chitosan-based nanoparticle platforms to overcome the limitations of traditional systems, offering improved sensitivity, earlier imaging time points, and enhanced diagnostic accuracy for infection and inflammation imaging. Therefore, the present findings reinforce the potential of ^99m^Tc–CDX–CS-NPs as a promising and efficient radiotracer for infection and inflammation imaging, outperforming several previously reported radiolabeled antibiotic formulations in terms of targeting efficiency and imaging contrast [73].

## Conclusion

The formulation of CDX–CS-NPs based on chitosan nanoparticles was characterized using transmission electron microscopy (TEM), X-ray diffraction (XRD), Fourier-transform infrared spectroscopy (FT-IR), zeta potential analysis, and UV–visible spectrophotometry. Radiolabeling was performed via a simple direct labeling approach, yielding ^99m^Tc–CDX–CS-NPs with a radiochemical yield of approximately 94%. The stability of the radiocomplex was assessed in vitro using fresh human serum, demonstrating adequate stability for up to 6 hours, which is sufficient for its application as an imaging agent for the localization of inflammatory foci. The in vivo biological evaluation of ^99m^Tc–CDX–CS-NPs in murine models of infection and inflammation revealed a strong ability to accumulate at both septic and aseptic inflammatory sites. High target-to-non-target (T/NT) ratios were observed, reaching 8.55 at 2 hours post-injection in infected (septic) mice and 6.02 at 2 hours post-injection in inflamed (aseptic) mice. Overall, the obtained results demonstrate that ^99m^Tc–CDX–CS-NPs possess considerable potential as a novel radiotracer for imaging acute inflammation and infection. The observed biodistribution profile and elevated T/NT ratios indicate effective localization at sites of pathological inflammation. However, the current study revealed a notable limitation, namely the inability of the radiotracer to definitively differentiate septic from aseptic inflammatory lesions. Although significantly greater uptake was observed in septic foci, suggesting enhanced accumulation in infectious processes, the degree of overlap between the two conditions limits its diagnostic specificity. Therefore, further investigations are warranted to elucidate the underlying uptake mechanisms and to optimize the formulation for improved discrimination between infectious and sterile inflammatory responses. Such studies may enhance the clinical utility of this radiotracer and represent a key direction for future work.

## Data Availability

All the data were collected at the Egyptian Atomic Energy Authority. All data supporting the findings of this study are available within the manuscript
